# Prevalence and Characteristics of Lung Involvement on High Resolution Computed Tomography in Patients with Ankylosing Spondylitis: A Systematic Review

**DOI:** 10.1155/2012/965956

**Published:** 2012-02-22

**Authors:** Abdellah El Maghraoui, Mohamed Dehhaoui

**Affiliations:** ^1^Rheumatology Department, Military Hospital Mohammed V, P.O. Box 1018, Rabat, Morocco; ^2^Statistics Department, Hassan II Agronomic Institute, Rabat, Morocco

## Abstract

To determine the prevalence of lung involvement and the spectrum of abnormalities revealed on HRCT in patients with AS, a systematic literature review was conducted in the Medline database up to May 2009 and in the abstracts of rheumatology scientific meetings (2006–2008). A hand search of references was also performed. Among the 264 selected articles, 10 articles (303 patients) allowed a calculation of the prevalence of lung abnormalities on thoracic HRCT in AS. A total of 185 patients (61%) had an abnormal thoracic HRCT: upper lobe fibrosis in 21 (6.9%), emphysema in 55 (18.1%), bronchiectasis in 33 (10.8%), and ground glass attenuation in 34 (11.2%). Non specific interstitial abnormalities were observed in 101 (33%) patients. The most common observed abnormalities were pleural thickening (52%), parenchymal bands (45%) and interlobular septal thickening (30%). Only the prevalence of upper lobe fibrosis increased significantly with disease duration (3 studies). Mild and non-specific interstitial abnormalities on thoracic HRCT are common in patients with AS, even in patients with early disease and without respiratory symptoms.

## 1. Introduction

Ankylosing spondylitis (AS) is a chronic rheumatic disorder characterized by inflammation of the enthesis (especially of the axial skeleton) and sometimes the joints, which may lead to ankylosis [1]. AS is a multisystemic disease and extraarticular features include uveitis, carditis, colitis, and osteoporosis [2–4]. Lung involvement is now a well-recognized extra-articular feature of AS. It was initially described in 1941 [5]. However, it was considered as an extra-articular manifestation of the disease only since 1965 [6]. The prevalence of pleuropulmonary involvement in AS varies considerably (0–30%) in the medical literature. The most frequently recognized pleuropulmonary manifestations are upper lobe fibrosis, mycetoma formation, and pleural thickening [7–12]. The advent of high resolution computed tomography (HRCT) in the mid 1980s has allowed physicians to examine the entire lung parenchyma and pleura in many conditions with diffuse lung disease using a noninvasive method [13].

The objective of this analysis was to determine the prevalence of lung involvement and the spectrum of abnormalities revealed on HRCT in patients with AS according to the disease duration and to describe its clinical characteristics by a systematic literature review.

## 2. Material and Methods

To identify relevant studies, allowing calculation of the prevalence of lung involvement in AS or to determine its characteristics or both, a systematic analysis of literature was performed in May 2009.

### 2.1. Literature Search Strategy

Publications were identified in the PubMed Medline database up to May 2009 and in the abstracts of EULAR (European League Against Rheumatism) and the ACR (American College of Rheumatology) scientific meetings of the years 2006, 2007, and 2008. Two searches were carried out in the Medline database which used the following key words: for the first search “spondylarthritis” or “ankylosing spondylitis” and “interstitial lung disease” or “apical fibrosis” (limits were English, French, humans); for the second search “spondylarthritis” or “ankylosing spondylitis” and “thoracic CT scan” (limits were English, French, humans). Original articles were selected if they reported the prevalence and/or the characteristics of lung involvement on thoracic HRCT in AS. The analysis concerned adults over 16 years old; studies concerning juvenile arthritis were excluded. Lastly, a hand search of references was also performed.

### 2.2. Data Collection

The articles were analysed by one author (A. El Maghraoui) according to a predetermined abstraction sheet. The following items were collected: number of AS patients (and criteria for the diagnosis), number of patients reported as having lung involvement on HRCT, sex, age at the moment of the study, disease duration, and characteristics of lung involvement on HRCT.

### 2.3. Statistical Analysis

Statistics were descriptive. The prevalence of lung involvement on HRCT was calculated as the number of patients with AS presenting one or more lesion on HRCT over the total number of patients with AS. Standard deviations and means were calculated based on available data.

## 3. Results

### 3.1. Article Selection

**Figure 1:**
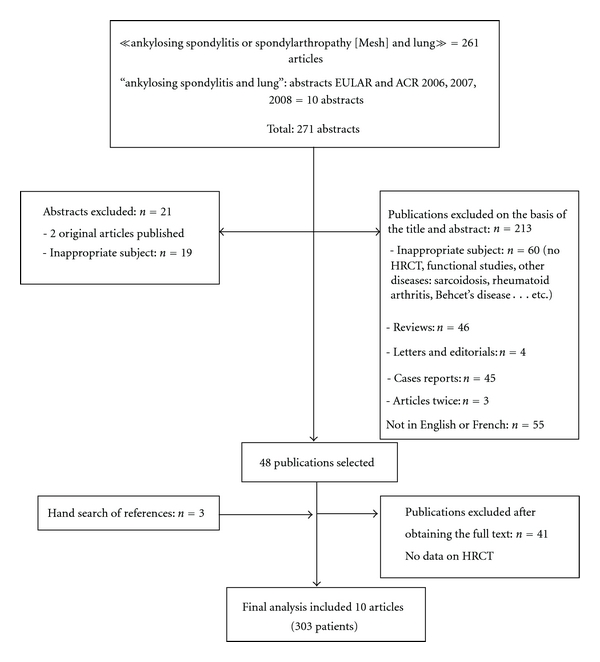
Flowchart summarizing the search process.

The Medline database and the congress abstracts search yielded 261 articles. With the hand search of references, a total of 10 articles were entered in the analysis (Figure 1). Main reasons for exclusion were reviews and case reports (42.7%), articles not related to AS or lung involvement on HRCT (28.1%).

### 3.2. Prevalence

To calculate prevalence, a total of 10 articles were assessed. All the studies were cross-sectional. The total number of patients with AS was 303. The diagnosis of AS was based on modified New York criteria for all the patients [14]. Of 303 patients, 258 (85.1%) were male. Mean (SD) age was 40.7 (6.2) years. Mean disease duration was 11.7 (5.2) years. Only 18 (5.9%) patients were reported to have pulmonary symptoms, 73 (24%) were current smokers and 41 (13.5%) ex-smokers.

A total of 185 patients (61%) was reported as having an abnormal thoracic HRCT imaging. Among these patients, upper lobe fibrosis were observed in 21 (6.9%), emphysema in 55 (18.1%), bronchiectasis in 33 (10.8%), and ground glass attenuation in 34 (11.2). Nonspecific interstitial abnormalities were observed in 101 (33%) patients. The most common observed abnormalities were pleural thickening (52%), parenchymal band (45%), and intrelobular septal thickening (30%) (Table 1).

Only 3 studies analyzed the prevalence of HRCT abnormalities according to disease duration. Among the 75 patients at risk, the prevalence of upper lobe fibrosis increased with disease duration (Table 2).

## 4. Discussion

This systematic literature review reports the prevalence and characteristics of chest HRCT abnormalities in AS. In this systematic analysis, the prevalence of pulmonary abnormalities in AS was high: 61% for a mean disease duration of 11.7 (5.2) years. Upper lobe fibrosis, with or without cavitation, was a rare HRCT finding reported in 6.9%. Prevalence increased with disease duration in the few studies where data were available. The main characteristic of pulmonary abnormalities in AS was the high frequency of mild nonspecific interstitial abnormalities. In this systematic analysis, parenchymal bands and sparse thickening of the interlobular septa, characterized as linear opacities, were the most frequent findings considered as nonspecific interstitial lung disease [15–26].

The interpretation of prevalence must take into account disease duration. However, few studies analyzed the prevalence of lung abnormalities on HRCT according to disease duration. Indeed, in this analysis the prevalence of upper lobe fibrosis in AS increased with the mean disease duration; this prevalence reached 21% for a mean disease duration >10 years. This was not the case for the other observed abnormalities. However, it should be noted that these analyses concerned mean disease duration, and not true categories of disease duration, which is one of the limitations of this work, which is unavoidable in systematic literature reviews.

Smoking-induced lung disease is a complex group of disorders, varying from the well-known entity of chronic obstructive pulmonary disease to more recently described interstitial lung diseases which may resemble to the reported abnormalities described here [27]. Among the studied patients in this systematic review, about 37% had a history of smoking. However, no differences in prevalence of HRCT lung abnormalities were noted between smokers and no smokers.

The main limit of this systematic literature review is a possible selection bias. None among the published studies had a control group. Seven studies included consecutive patients with AS without history of pulmonary symptoms and excluded patients with history of tuberculosis, earlier pneumonia, and exposure to dust or inhaled gases, while 2 studies included patients even when patients had pulmonary symptoms and one study selected patients with no history of smoking. With regard to the evaluation of CT abnormalities, another limitation is that different scanners were used which may have hampered precise evaluation of subtle lung changes. The analysis of images generated with different reconstruction algorithms may have affected the detection of mild ground-glass opacity.

The effect of anti-TNF blockers on interstitial lung fibrosis and other abnormalities has never been evaluated. However, this issue may become even more important as these agents are now widely prescribed for AS [28, 29]. Moreover, many recent case reports described contrasting effects on pulmonary fibrosis in patients with rheumatoid arthritis (RA). Ostor et al. [30] reported fatal exacerbation of RA-associated fibrosing alveolitis in three patients receiving infliximab. On the other hand, Bargagli et al. [31] described a beneficial effect of infliximab in the treatment of RA associated with interstitial lung disease.

The administration of anti-TNF agents is associated with an increased risk of reactivation of latent tuberculosis even though the measures recommended to screen and to prevent this reactivation have been shown to be effective [32]. Thus, in clinical practice thoracic HRCT may be useful to identify a suspicious abnormality in chest X-rays, especially when anti-TNF therapy is planned. Clinicians must be aware of the HRCT abnormalities observed in AS, which must not be confounded with tuberculosis lesions.

In summary, this systematic review revealed a great percentage of defined as well as mild and nonspecific interstitial abnormalities on thoracic HRCT undetectable on plain radiography in patients with AS. These lesions are common even in patients with early disease and without respiratory symptoms. The significance of such changes is unknown and must await prospective longitudinal studies to determine their natural history. Among the observed lung abnormalities, only apical fibrosis seemed to be more prevalent with increasing disease duration.

## Figures and Tables

**Table 1:** Prevalence of thoracic HRCT abnormalities in patients with AS.

	Casserly et al. [15]	Turetchek et al. [17]	Senocak et al. [18]	Kiris et al. [19]	El Maghraoui et al. [20]	Souza et al. [21]	Altin et al. [22]	Ayhan-Ardic et al. [23]	Sampaio-Barros et al. [24]	Baser et al. [25]	Total: *n* (%)
*N*	26	21	20	28	55	17	38	20	52	26	303
Prevalence of abnormalities on HRCT	18	15	17	18	31	15	27	10	21	13	185 (61)
Apical fibrosis	2		3		5	1	5	2	1	2	21 (6.9)
Emphysema	4	2	9		5	6	13	2	5	9	55 (18.1)
Brochiectasis	6	2	3	2	4	2		2	4	8	33 (10.8)
Ground glass attenuation	2	1	6	2	2		14			7	34 (11.2)
Non specific lung interstitial abnormalities	11	6	9	10	26	10	19		10		101 (33.3)
Lymphadenopathy	3								6		9 (2.9)
Pleural thickening	1	6	9		13	3	13		4	4	53 (17.4)
Micronodules	1	2	8	7	7	1			3	4	33 (10.8)
Pulmonary cysts						3			1		4 (1.3)
Bronchial wall thickening	4	6		2			12				24 (7.9)
Sub-pleural band	6				6						12 (3.9)
Parenchymal band	8		3	5	13		14			3	46 (15.1)
Irregular interfaces	3				4						7 (2.3)
Blebs	3				7						10 (3.3)
Intrelobular septal thickening	7	7		1			16				31 (10.2)
Linear septal thickening		6	9							6	21 (6.9)
Pleural tags	4										4 (1.3)

**Table 2:** Prevalence of thoracic HRCT abnormalities in patients with AS according to disease duration.

Disease duration	<5 yrs	5–10 yrs	>10 yrs	*P*
Number of patients at risk	50	18	23	
Ground glass attenuation	9 (18)	2 (11.1)	4 (17.3)	0.855
Upper lobe fibrosis	3 (6)	2 (11.1)	5 (21.7)	0.049
Brochiectasis	9 (18)	1 (5.5)	5 (21.7)	0.881
Emphysema	11 (22)	3 (16.6)	8 (34.7)	0.309
